# Perceptions of Mental Health, Shame and Help‐Seeking Among Sikhs in the UK: A National Survey

**DOI:** 10.1002/jcop.70043

**Published:** 2025-09-12

**Authors:** Supreet Uppal, Opinderjit Takhar, Ranjit Khutan, Niall Galbraith

**Affiliations:** ^1^ School of Psychological, Social and Behavioural Sciences Coventry University Priory St Coventry UK; ^2^ Faculty of Education, Health and Wellbeing, MC Building University of Wolverhampton Wolverhampton UK

**Keywords:** community, external shame, family, help‐seeking, internal shame, mental health, shame and help‐seeking, Sikhs Sikh mental health

## Abstract

To understand UK Sikh community's perceptions of mental health shame, their attitudes towards mental health causes, treatments and help‐seeking and the characteristics associated with these perceptions. An anonymous, online survey collected views from 1001 Sikh respondents on the causes and treatments for mental health (MH) difficulties and perceived barriers to help‐seeking. The Attitudes to Mental Health Problems (ATMHP) questionnaire also measured respondents' perceptions of external, internal and reflected mental health‐related shame. Respondents mostly endorsed biopsychosocial causes of MH difficulties and most viewed psychology/psychiatry/counselling, social and spiritual support as the main treatments. Shame, stigma and nonacceptance were seen as barriers to seeking help. Compared to previous UK surveys using the ATMHP, our Sikh respondents scored more strongly on most dimensions of MH shame compared to the general population or to students; they tended to score similarly or higher on MH shame compared to a previous ATMHP survey of UK South Asians. Internal shame was higher in single, younger people. Those inclined to help others perceived more negative MH attitudes within their community. Higher internal shame, higher perceived external family shame, and higher perceived negative community attitudes to MH were found in those who had had previous MH problems themselves. Within this generally young sample, perceptions of MH shame within communities/families were quite high, as was internalised shame. Such attitudes pose a significant barrier to MH help‐seeking. These findings highlight the need for culturally sensitive MH services for the UK Sikh population.

## 1 Introduction

In the UK, South Asian (SA) communities are culturally diverse and heterogeneous. There is a considerable range in languages, religions, dietary practices and migration histories within the UK SA population (Anand and Cochrane 2005). The higher prevalence of mental health (MH) difficulties in the British SA community (see Fazil and Cochrane 1998; Nazroo 2003; Sonuga‐Barke and Mistry 2000; Weich et al. 2004), compared to the general population, has been highlighted as a concern by the UK's Department of Health (Department of Health 2011). This high prevalence has been attributed to inequalities in health care settings (e.g., Moller et al. 2016).

Acculturation is another potential explanation for high MH prevalence in the UK SA population. Although some evidence shows that immigrants adapt well in their new intercultural settings (Motti‐Stefanidi and Masten 2017), other research shows immigrants are at increased risk for psychological distress (Dimitrova and Aydinli‐Karakulak 2016; Harper 2001; Nakash et al. 2012; Shoshani et al. 2016). Acculturative stress can hinder development of a stable cultural identity, particularly in second generation immigrants, in turn affecting the prevalence of MH difficulties (Bhugra 2004; Bhui et al. 2005). South Asian immigrants can experience MH difficulties upon arrival to a new country due to migration experiences (Berry and Sam 1997; Rudmin 2009; Sam and Moreira 2012). Panjabi women who had emigrated from India to the USA, report higher depression and anxiety—particularly by those women who did not speak English, with limited educational/employment opportunities, lack of social support, isolation and loneliness (Balan and Mahalingam 2015; Roberts et al. 2016). South Asian women can also experience acculturative stress due to intergenerational conflict within the household (Samuel 2009).

A further crucial factor influencing the prevalence of MH difficulties across the UK is stigma. Phelan et al. (2015) defines stigma as a mark of disgrace that sets individuals apart and recognises them as being different from others. When an individual is labelled by their illness, they tend to be stereotyped into a group and are no longer seen as an individual (Angermeyer and Matschinger 2003; Sjöström 2018).

Culture beliefs play a vital role in influencing social stigma towards people with MH difficulties (Roberts et al. 2016; Van Brakel 2006). In some immigrant communities, mental health is seen as a Western cultural construct and some cultures may not conceptualise some symptoms as psychological disorders (Kleinman 1987; Vaillant 2012). For example, there are no definitions for terms such as ‘mental well‐being’ or ‘mental distress’ in *Gurmukhi* nor Panjabi (Bhugra 2004). In Western countries, compared to the general population, south Asian communities may be more likely to use supernatural and religious explanations for MH difficulties (Fabrega 1995; Lauber and Rössler 2007). However, in younger South Asian samples, with higher numbers of professional and financially stable respondents, culturally traditional beliefs about MH might be less prevalent than in older generations (Tabassum et al. 2000).

UK studies report that family reputations and marriage concerns heavily influence the management of MH difficulties within SA communities (Tabassum et al. 2000). There may be particular pressure to control emotional expression, with the risk of family disrepute if help is sought outside of the family. Evidence suggests that some cultural and religious beliefs about the causation of MH can lead to shame and blame (Bradby et al. 2007; Hatfield et al. 1996; Knifton 2012). Compared to the general population, UK South Asian respondents show higher levels of external shame in relation to mental health—That is, shame based on what other people think (Gilbert et al. 2007), but similar levels of internal shame (self‐directed shame). This often results in ethic minorities hiding any difficulties related to MH. According to Bradby et al. (2007), such shame in the South Asian community perhaps stems from the notion that MH difficulties are genetically inherited. Therefore, association with MH difficulties tarnishes the entire family's reputation. This is consistent with Irving Goffman's (1963) concept of ‘spoiled social identity’, whereby those with stigmatised traits are set apart and devalued. More than 40 years later, Gilbert et al. (2007) also describe the concept of ‘reflected’ shame—the perception that one has disgraced one's family or community by not living up to expectations (see also Gehlot and Nathawat 1983).

The shame attached to help‐seeking for mental health problems can be understood with reference to social rank theory (e.g., Gilbert 1998; Price 1972). Gaining social approval has been highly adaptive during human evolution and thus having qualities which others (not only ourselves) value highly, contributes to our self‐esteem. The negative connotations around mental health help‐seeking can give rise to worry over whether such help‐seeking will lower one's value to others and thus threaten one's social rank. Being socially devalued in this way can result in external shame—the negative affect which results from disapproval, rejection or put‐down from others. This kind of shame is externalised as it is a perception of how others see the self. As our self‐esteem depends on approval from others, shame from others can easily become *internalised*, such that the beholder adopts the same disapproval of the self (Gilbert 1998).

As a result of stigma and shame, people in South Asian communities are less likely to seek help from MH services and might rely more so on social, cultural or religious sources of support (Lee et al. 2009; Leung et al. 2012). Numerous studies in the UK and USA have recommended that existing MH services should be more sensitive to the nuanced relationships between shame, stigma and MH in South Asian communities (Gilbert et al. 2007; Gill 2010; Lee et al. 2009; Roberts et al. 2016). However, although various studies have been conducted on MH attitudes in the South Asian populations in the UK or US, very few have focused specifically on Sikhs. Although Sikh culture has many similarities to other SA communities, there are important cultural differences between these communities also. The Sikh culture holds different notions of social equality, than that of the Hindu tradition for example, where the caste system serves as a strong basis for social hierarchy (Singh 1989).

In the UK, the Sikh community represents 0.88% (*N* = 525,865) of the England and Wales population (Office for National Statistics [ONS], 2021). Research on MH in the British Sikh community and the barriers they face in seeking help, is represented inadequately within the literature. This results in the underdevelopment of services tailored for them. The current study aims to understand perceptions of mental health shame in Sikhs living in the UK (hereafter referred to as UK Sikhs), their attitudes towards mental health causes, treatments and help‐seeking and the characteristics associated with these perceptions. In pursuit of these aims, the following hypotheses as well as more exploratory research questions, have been formulated.

### 1.1 Hypotheses

1. UK Sikhs will have stronger perceptions of MH‐related shame compared to previous normative data from the UK general population and previous surveys of UK South Asian respondents.
2. In accordance with social rank theory, having had previous mental health problems will be associated with internal shame.
3. In accordance with social rank theory, our own self‐esteem depends on our perception of how others see us. Thus, the relation between previous mental health problems and internal shame will be partly explained (mediated) by external shame from community/family.
4. Also in line with social rank theory: the relation between previous mental health problems and internal shame will be mediated *more strongly* by externalised mental health shame from community/family than by awareness of negative mental health attitudes in the community/family.

### 1.2 Research Questions

1. In UK Sikhs, which demographic characteristics and previous MH experiences are associated with perceptions of (internal, external and reflected) MH shame?
2. What are UK Sikhs' views on the causes of MH difficulties, the most effective treatments for MH difficulties and the main barriers to receiving help for MH difficulties?

## 2 Materials and Methods

### 2.1 Participants

Participants' characteristics are shown in Table 1. The sample comprised UK Sikh adults and was a relatively young one, with just over half of respondents being in the 18–25 age group. Most were single, spoke English as their primary language, were born in England and had been university educated. Various forms of recruitment took place such as internet advertisement on social media and popular online Sikh forums, posters in Gurdwaras, and contacts through the Centre for Sikh and Panjabi Studies (based in the authors' university). Participants were not paid.

**Table 1:** Part 1. The characteristics of the Sikh participants (*N* = 1001).

	Frequencies	%
Sex		
Males	395	39.5
Females	604	60.3
Prefer not to say	2	0.2
Age group		
18–25	525	52.4
26–35	248	24.8
36–45	150	15.0
46–55	54	5.4
56–56	12	1.2
76+	1	0.1
Prefer not to say	11	1.1
Marital status		
Single	652	65.1
Married	282	28.2
Divorced	30	3.0
Widow	1	0.1
Separated	12	1.2
Prefer not to say	5	0.5
Other	19	1.9
Place of birth		
England	844	84.3
Scotland	20	2.0
Europe	7	0.7
India	75	7.5
Africa	16	1.6
Other	39	3.9

Part 2. The characteristics of the Sikh participants (*N* = 1001)		
	Frequencies	%
Region of residence		
North East	14	1.4
North West	20	2.0
Yorkshire and The Humber	45	4.5
East Midlands	93	9.3
West Midlands	453	45.3
East of England	14	1.4
London	223	22.3
South East	89	8.9
South West	24	2.4
Scotland	22	2.2
Northern Ireland	1	0.1
Wales	3	0.3
Highest level of education		
Primary school	1	0.1
Secondary school	37	3.7
Sixth form/college level	234	23.4
Bachelor's degree or equivalent	504	50.3
Master's degree or equivalent	173	17.3
Doctoral or equivalent	20	2.0
Post‐doctoral or equivalent	6	0.6
Prefer not to say	17	1.7
Other	9	0.9
Primary language		
English	861	86.0
Panjabi	130	13.0
Other	10	1.0
Sikh identity		
Amritdhari	221	22.1
Kesdhari	181	18.1
Sehajdhari	546	54.5
Prefer not to say	47	4.7
Other	6	0.6

Part 3. The characteristics of the Sikh participants (*N* = 1001)		
	Frequencies	%
Sikh identity		
Amritdhari	221	22.1
Kesdhari	181	18.1
Sehajdhari	546	54.5
Other	47	4.7
Prefer not to say	6	0.6
Gurdwara attendance		
Daily	53	5.3
More than once per week	155	15.5
Weekly, not always Sunday	179	17.9
Every Sunday	68	6.8
Monthly	225	22.5
Less than monthly	276	27.6
Prefer not to say	22	2.2
Other	23	2.3
Are you able to offer help to anyone?		
Yes	819	81.8
No	148	14.8
Prefer not to say	34	3.4
Have you experienced MHD?		
Yes	658	65.7
No	324	32.4
Prefer not to say	19	1.9
Did you seek professional help?		
Yes	339	33.9
No	483	48.3
Prefer not to say	22	2.2
What other support: faith/spiritual?		
Yes	453	45.3
No	548	54.7

### 2.2 Data Analysis

#### 2.2.1 Multinomial Logistic Regressions

All data analysis employed the Statistical Package for Social Science (SPSS), Version 29. Multinomial logistic regressions tested relationships between predictors and categorical criterion variables. Predictor variables were the seven subscales of the ATMHP (Community attitudes to MH, family attitudes to MH, community external shame, family external shame, internal shame, family‐reflected shame, self‐reflected shame). The criterion variables were (1) marital status (separated/divorced/widowed), (2) age group (18–25, 26–35, 36‐45, 46+), (3) Sikh identity (Amritdhari—Initiated into Khalsa, Kesdhari—Sikhs who do not cut their hair, but are not initiated, Sehajdhari—Sikhs who cut their hair), (4) Primary language (English, Punjabi), (5) Frequency in attending the Gurdwara (daily, more than once a week, weekly not always Sunday, every Sunday, monthly, less than monthly), (6) Are you able to offer help to anyone experiencing any kind of mental health difficulties? (yes, no), (7) Have you experienced any kind of mental health difficulties? (yes, no), (8) Did you seek any professional help for your mental health difficulties? (yes, no), (9) What other support did you seek for your mental health difficulties: faith/spiritual? (selected, not selected).

Multinomial logictical regression provides data on which predictor has a significant relationship with the criterion (or outcome) variable (Field 2009). Relationship effect size can be expressed as an odds ratio, reflecting the odds that the predictor variable is higher or lower in one category of the criterion compared to another category. So, for example, the odds that internal shame might be higher in single people compared to married people.

#### 2.2.2 Mediation Analyses

Mediation analysis with bootstrapping was conducted using the Hayes macro for SPSS (Hayes 2012; Preacher and Hayes 2004). Here, the aim was to examine whether the relation between previous mental health problems (predictor variable) and internal shame (outcome variable) could be explained fully or partly by the strength of other variables (mediators), namely external community‐related shame, external family‐related shame, negative community attitudes and negative family attitudes. In other words, a relation between previous mental health problems and internal shame is hypothesised, but can this relationship can be understood more fully by testing for indirect relationships between these two via the mediatiors? Bootstrapping involves sampling repeatedly form the data to estimate the relationships between variables.

### 2.3 Measures

#### 2.3.1 Demographic Characteristics

The first part of the online questionnaire presented demographic questions: Age (18–25, 26–35, 36–45, 46–55, 56–65, 65+); Sex; martial status (single, married, separated, divorced, widowed); region of residence in the UK (Scotland, Northern Ireland, Wales, North East, North West, Yorkshire & The Humber, East Midlands, West Midlands, East of England, London, South East, South West); place of birth (Scotland, England, Wales, Nortrhern Ireland, Europe, India, Africa, other); highest level of education; Sikh identity (Amritdhari (Initiated into Khalsa), Kesdhari (Do not cut hair, but not initiated), Sehajdhari (Sikhs who cut their hair)); and frequency of visits to the Gurdwara (Daily, more than once a week, weekly not always Sunday, every Sunday, monthly, less than monthly).

#### 2.3.2 Mental Health Beliefs and Experience

The second part of the questionnaire asked questions on previous experience of and beliefs about MH difficulties. These questions were devised by the authors inorder to answer research question 2: are you able to offer help to anyone experiencing mental health difficulties? (yes, no); have you experienced any kind of mental health difficulties? (yes, no); did you seek any professional help for your mental health difficulties? (yes, no); did you seek mental health help through faith? (yes, no); what causes mental health?; who can help treat mental health conditions?; what are the main barriers to receiving help for mental health conditions?; what is the diagnosis or the nature of the condition or symtoms you experienced?

#### 2.3.3 Attitudes Towards Mental Health Problems (ATMHP)

The remaining part of the questionnaire measured attitudes around the shame of MH and help‐seeking. The ATMHP is a 35‐item questionnaire with, seven subscales (Gilbert et al. 2007). The questionnaire recognises that there are different dimensions to MH shame: one's perception of how others might see them, one's perception of themselves and one's perception of bringing shame upon others. The first subscale (Community Attitudes) measures one's perceptions of community's attitudes to MH problems (four items, e.g., *My community sees mental health problems as a personal weakness*). The second subscale (Family Attitudes) measured perceptions of how one's family views MH problems (four items, e.g., *My family see mental health problems as something to keep secret*). The third subscale (External Shame—Community) measures one's perception of how their community would view *them* if they had a MH problem (five items, e.g., *I think my community would look down on me*). The fourth subscale (External Shame—Family) measures perception of how one's family would view *them* if they had a MH problem (five items e.g., *I think my family would see me as inferior*). The fifth subscale (Internal Shame) measures self‐evaluation if one were to have a MH problem (five items, e.g., *I would see myself as a failure*.). The sixth subscale (Family Reflected Shame) captures perceptions of how one's famly would be shamed if one were to have MH problems (seven items, e.g., *My family would be blamed for my problems*). The seventh subscale (Self‐Reflected Shame) measures perceived shame on oneself if a close family member were to have MH problems (five items, e.g., *I would worry that others would look down on me*).

Higher scores on the ATMHP reflect more negative attitudes. The responses are scored on a four point scale (0—do not agree, 1—agree a little, 2—mostly agree, 3—completely agree). The ATMHP has demonstrated good internal reliability (Gilbert et al. 2007; Hampton and Sharp 2014).

The data and the questionnaire can be accessed via the Open Science Framework https://osf.io/cg845/?view_only=b4b493c5008a49b6b1307c8e2d43e737.

### 2.4 Procedure

The survey was created through Online Survey software developed by the authors' University. Ethical approval was granted by the host University's Faculty of Arts Ethics Committee (Ref 2018/19:11). Snowball and opportunistic sampling (Baltar and Brunet 2012) was employed by approaching large Sikh organisations, communities and places of worship. The online survey was advertised on several different platforms such as Twitter, Facebook and Instagram, through online forums, posters at local Gurdwaras, and through radio and TV channels. A list was compiled of Sikh organisations that interacted with large populations of Sikhs and these organisations were contacted by telephone.

Study information and consent pages were uploaded on the software explaining the intention of the survey, what would be studied, length of time expected to participate and whom to contact for any queries. Once online consent was received, the survey took approximately 10–15 min to complete. A debrief document appeared following the survey's end. All responses were anonymouys and no IP addresses were collected. At the start, participants were made aware of the option to withdraw from the research.

## 3 Results

Responses to questions about demographic characteristics and mental health experience are shown in Table 1. Firstly, we describe the frequency of views about the causes, views on who can help to treat MH difficulties and views on the main barriers to receiving help for MH difficulties. Psychosocial factors (including family) and physical factors are seen by approximately 80% or more as the main causes of MH difficulties. Psychology/psychiatry/counselling was the most commonly endorsed treatment option, with a majority of the sample also seeing friends and family and spititual help as important. Shame, stigma and acceptance (lack of) were endorsed by most as barriers as was awareness of services; pragmatic barriers (cost, availability and inconvenience) were somewhat less commonly endorsed. The nature of MH conditions previously experienced: anxiety, depression and stress‐related conditions/symptoms were most commonly reported.

The current sample of Sikh respondents was compared to normative data on the ATMHP, specifically from Gilbert et al. (2007) where the sample comprised both UK‐Asian respondents and UK non‐Asian respondents and Kotera et al. (2021) whose sample comprised UK university students studying business (see Table 2). As can be seen from Table 2, the Sikh sample in the current study scored significantly higher than the Gilbert et al. (2007) UK non‐Asian sample on all dimensions of shame apart from one (internal shame). Our Sikh sample also scored significantly higher than Kotera et al.'s (2021) UK student sample on four dimensions of shame but lower on external family shame. Our Sikh sample tended to score higher or similarly to Gilbert et al.'s (2007) UK‐Asian sample, but were significanly higher on perceptions of negative community attitudes to MH.

**Table 2:** ATMHP Means and standard deviations (SD). Current sample compared to samples from two other previous studies.

Variable (range of possible scores)	Sikh (current study)	UK‐Asian (Gilbert et al. 2007)	UK non Asian (Gilbert et al. 2007)	UK business students (Kotera et al. 2021)
	Mean SD *N* = 1001	Mean SD *N* = 89	Mean SD *N* = 94	Mean SD *N* = 274
Comm att	7.15	**5.19** **	**3.79** **	**2.92** **
(0–12)	(3.34)	(3.33)	(2.74)	(3.36)
Family att	3.76	3.11	**2.06** **	3.64
(0–12)	(3.70)	(3.13)	(2.79)	(3.56)
Ext Com shame	7.97	7.03	**5.51** **	**4.10** **
(0–15)	(4.64)	(4.34)	(3.88)	(4.50)
Ext fam shame	4.00	3.80	**2.52** **	**5.35** **
(0–15)	(4.75)	(4.24)	(4.14)	(4.30)
Int shame	7.00	7.31	6.66	**4.65** **
(0–15)	(4.61)	(4.62)	(4.57)	(4.30)
Fam ref shame	8.46	9.21	**6.03** **	**6.30** **
(0–21)	(6.00)	(6.07)	(5.24)	(5.88)
Self ref shame	4.33	3.18	**2.71** **	4.45
(0–15)	(4.53)	(4.36)	(3.50)	(4.95)

** Indicates significant comparison ≤ 0.001. Bold means indicate a statistically significant mean difference from our Sikh sample.

### 3.1 Associations With Shame

A series of multinomial logistic regressions were computed to establish whether perceptions of the shame of mental health were associated with demographic characteristics, previous experience of mental health and help‐seeking. Nine multinomial logistic regressions were conducted, therefore, to protect against family‐wise error (inflated chances of finding spurious significant results), a Bonferroni correction was applied to alpha (0.05/9 = 0.0056). Hence, only *p* values < 0.006 were deemed statistically significant. The significant logistic regressions are reported here.

#### 3.1.1 Internal Shame

Those who would feel higher levels of internal shame about having MH problems were less likely to be married than single (odds ratio = 0.95; *b* = −0.05, standard error (SE) = 0.02, Wald *χ* ^2^(1) = 8.35, *p* = 0.004) and were less likely to be separated/divorced (OR = 0.89; *b* = −0.12, SE = 0.04, Wald *χ* ^2^(1) = 8.89, *p* = 0.003). Those with stronger internal shame were also less likely to be in the 46+ group (OR = 0.84; *b* = −0.18, SE = 0.04, Wald *χ* ^2^(1) = 23.85, *p* < 0.001) than in the 18–25 group. Those who would feel higher levels of internal shame were more likely to say that, they themselves, had had previous MH problems (OR = 1.07; *b* = 0.07, SE = 0.02, Wald *χ* ^2^(1) = 13.94, *p* < 0.001).

#### 3.1.2 Family Related Shame

Those who perceived higher external shame from family, were also more likely to say they themselves had had previous MH problems (OR = 1.13; *b* = 0.13, SE = 0.03, Wald *χ* ^2^(1) = 19.29, *p* < 0.001).

#### 3.1.3 Community Related Shame

Those who perceive more negative attitudes to MH within their community, were more likely to say they would be able to offer help to someone else with MH problems (OR = 1.16; *b* = 0.15, SE = 0.04, Wald *χ* ^2^(1) = 11.45, *p* < 0.001), were also more likely to say they themselves had had previous MH problems (OR = 1.12; *b* = 0.11 SE = 0.33, Wald *χ* ^2^(1) = 11.64, *p* < 0.001), were more likely to report having previously sought help for their own MH problems (OR = 1.12; *b* = 0.11, SE = 0.03, Wald *χ* ^2^(1) = 10.80, *p* = 0.001) and were more likely to say they had sought help for MH problems through faith (OR = 1.10; *b* = 0.10, SE = 0.03, Wald *χ* ^2^(1) = 10.23, *p* = 0.001).

Compared to the 18–25 group, the 26–35 group were more likely to perceive higher external shame from their community if they were to have a MH problems (OR = 1.11; *b* = 0.10, SE = 0.03, Wald *χ* ^2^(1) = 12.70, *p* < 0.001).

### 3.2 Mediation Analysis: The Relationship Between Previous Experience of Mental Health Problems and Internal Shame

**Figure 1:**
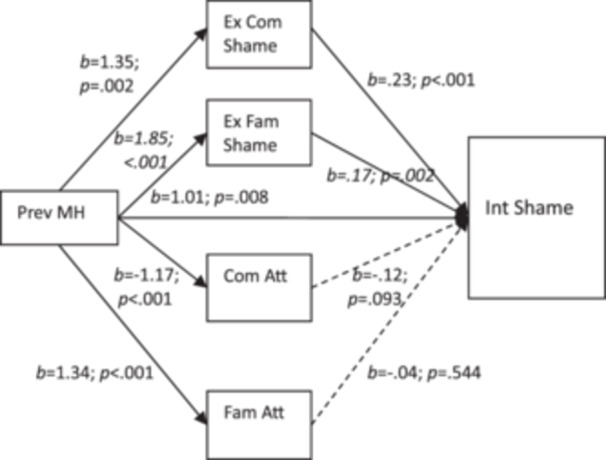
The mediator effects showing previous mental health problems (Prev MH) as a significant predictor of internal shame (Int Shame), mediated via both external community‐related shame (Ex Com Shame) and external family‐related shame (Ex Fam Shame) but not via negative community attitudes (Com Att) nor negative family attitudes (Fam Att). Unstandardised *b* weights shown for each pathway.

It was predicted that having had mental health problems would predict internal shame but that this relationship would be partly mediated (explained) by the presence of external shame. The relationship (simple total effect) between previous mental health problems and internal shame was significant (*b* = 1.44, *t* = 3.70, *p* < 0.001, 95% CI, 0.67 to 2.20). The full model, including the predictor and all mediators was significant (*F*(5, 813) = 17.93, *R* ^2^ = 0.10, *p* < 0.001) and in this model, the direct effect of previous mental health problems remained significant (*b* = 1.01, *t* = 3.70 *p* = 0.008, 95% CI, 0.27 to 1.75). There were two significant indirect effects (partial mediations) of the previous mental health problems→internal shame relationship. Those who have had previous mental health problems, report higher internal shame, but this is partly explained by how strongly they feel external community‐related shame (*b* = 0.30, *b*SE = 0.12, 95% CI, 0.11 to 0.56) and external family‐related shame (*b* = 0.32, *b*SE = 0.12, 95% CI, 0.10 to 0.58). Neither perception of negative community attitudes (*b* = −0.12, *b*SE = 0.09, 95% CI, −0.34 to 0.03) nor perception of negative family attitudes to mental health problems (*b* = −0.06, *b*SE = 0.10, 95% CI, −0.26 to 0.14) mediated the previous mental health problems→internal shame relationship (all pathways are shown in Figure 1 with unstandardised beta values).

## 4 Discussion

This is the first large (i.e., national with *n* > 1000) survey of shame‐related MH attitudes amongst the Sikh population in the UK. Our respondents mostly had a biopsychosocial view of the causes of MH difficulties and mostly viewed psychology/psychiatry as well as social and spiritual support as the principal treatments for MH difficulties. Although (lack of) awareness of services was endorsed by many as a barrier to help‐seeking, psychosocial factors such as shame, stigma and nonacceptance by others were seen as barriers to seeking help, more so than pragmatic reasons (e.g., cost, availability and inconvenience).

Our Sikh respondents scored significantly higher on most dimensions of the ATMHP, compared to previous studies on the UK general population or UK students (Gilbert et al. 2007; Kotera et al. 2021). Compared to a previous ATMHP survey of a UK South Asian sample (Gilbert et al. 2007), our Sikh respondents scored similarly on all dimensions of the ATMHP, bar one: they had notably higher perceptions of negative community attitudes compared to Gilbert et al.'s (2007) UK South Asian sample which included Muslims, Hindus and Sikhs.

The findings reveal which characteristics are associated with internal shame, shame pertaining to the family and shame pertaining to the community. Internal shame was more likely to be found in single, younger people and in those who had had previous MH problems. Those who perceived higher external shame from family if they themselves were to have MH problems, were also more likely to have had previous MH problems. Perceptions of negative community attitudes to MH were more likely in those who say they could help someone else with MH problems, in those who have had previous MH problems and those who report previously seeking‐help for their MH problems. Perceptions of external community shame in the event of MH problems were more likely in those in their 20s/30s. This is consistent with previous studies showing the importance of relational (family/community) factors in mental health and shame (see Bradby et al. 2007; Tabassum et al. 2000; Wang et al. 2018).

These findings suggest that shame is felt particularly by those who have lived through personal experience of MH problems. That is, awareness of stigmatising community attitudes and the sense of shame emanating from family may become more attuned when one has lived through the experience of MH difficulties and the alienation that can accompany this (see also Dolezal 2022). Our data reveal however, that having had this experience makes one more inclined to want to help others who may be experiencing MH difficulty. Suggesting that these experiences might enhance understanding and empathy for others enduring similar difficulties (see also Furnham and Sjokvist 2017).

Consistent with hypotheses and with social rank theory (Gilbert 1998, 2000), those who had had mental health problems reported stronger internal shame. As social rank theory argues that our own self‐esteem depends on our perceptions of how others see us, we predicted that the relation between having previous mental health problems and internal shame, would be partly explained by the perception that community/family would look down on ‘me’ if I had mental health problems. The mediation analyses supported this prediction and are consistent with Gilbert et al. (2007). Furthermore, and again in line with predictions and with social rank theory, the link between previous mental health problems and internal shame was *not* mediated by the mere perception of negative mental health attitudes in the community/family. One may be aware that such attitudes exist within the community/family, but they do not necessarily reflect how others see ‘me’ and thus do not explain internal shame as strongly.

Future studies should seek to further elucidate the causal link between external and internal shame in the Sikh population. For example, there is evidence that self (or internalised) stigma may emerge following exposure to public stigma (Vogel et al. 2013). Thus, it is possible that shame projected from community/family leads to personal internalisation of these beliefs. Research suggests that the internalising of public stigma—that is, stigma projected from others to the self—may be interrupted by instilling self‐compassion (Heath et al. 2018). This represents an opportunity for future research, where experimental or longitudinal studies may test these causal processes within the Sikh community.

Our respondents' conceptions of MH were generally consistent with contemporary psychological and medical science: the sample in the current study overwhelmingly endorsed psychosocial causes for MH difficulties as well as physical causes. Their view of treatments for MH difficulties also favoured psychological/psychiatric treatments as well as social and spiritual support, beliefs which are consistent with the contemporary evidence base (Koenig 2009; Thoits 2011). This indicates that our sample was psychologically literate. The majority (66%) had experienced MH difficulties themselves and most (82%) would help others with MH difficulties. This illustrates that the attitudes of our sample did not reflect the negative attitudes many of them perceived to be prevalent within their community. What this study does not do and which future research might aim for, is to establish whether an older sample from the Sikh community also holds similar attitudes to MH that our young, well‐educated sample does.

The importance of community and family attitudes and their effect on the shame associated with MH and MH help‐seeking is consistent with previous research on Sikh respondents from the US (Roberts et al. 2016) and Canada (Gill 2010). These findings suggest that changing community attitudes toward MH is essential to foster greater utilisation of MH servies by those in the Sikh community. Bradby et al. (2007) propose that the stigma of MH problems in British Asian families reflects Goffman's (1968) notion of ‘spoiled identity’, whereby it is the family whose identity risks being spoiled by association with MH problems. According to Bradby et al. (2007), this is particularly so when MH problems are conceptualised as inherited, reflecting negatively on families who may be seen as the carriers of such genes.

This perhaps presents an opportunity for MH literacy or other educational interventions, whereby debunking stigmatised misconceptions about the nature of MH problems may improve communities' understanding of the biopsychosocial causes of and treatments for psychological disorders— understandings which do not equate to shame or to spoiled reputations. Previous research has recommended the development of community psycho‐education on the causes and symptoms of psychological disorders (Thompson 2010), and previous research has suggested that improving knowledge of psychological disorders in Sikh communities can help to dispel myths about MH problems (Gill 2010).

The current findings suggest that mental health services, including hospital and inpatient but particularly community‐based services, should be designed to accommodate the MH beliefs of UK Sikhs to improve MH help‐seeking in this community. In particular, services should be sensitive to the important role that community/family‐related shame plays in MH for UK Sikhs and how these factors may inhibit help‐seeking. Consistent with this view, Bradby et al. (2007) suggest that outpatient MH services be located at distance from communities to aid confidentiality. Services might prioritise discretion and confidentiality in this way but perhaps a more sustainable approach would be to engage Sikh communities to codesign community‐based services to engender a sense of ownership and empowerment. This might involve incorporating traditional healing practices into service provision or other culturally relevant interventions, thus enhancing acceptability and effectiveness of services and potentially reducing stigma (see Cheng and Yen 2021; Jimenez et al. 2012). Others have argued (see Mann and Kaur 2025) that although cultural competence in mental health professionals is important, it is the design and delivery of services by community ambassadors which will help engender trust within ethnic minority communities such as UK Sikhs. Examples of services such as this in the UK are the Sikh organisations Taraki (Taraki, n.d.) and Sikh Your Mind (Sikh Your Mind n.d.), in which culturally‐authentic mental health awareness is developed within communities by disseminating people's experiences and stories through online media and via community events in conjunction with research. In keeping with this ethos, the authors will disseminate the current findings to the UK Sikh community via both online and in‐person mental health help‐seeking workshops, as part of our ongoing efforts to deliver research that has real‐life impact on communities.

### 4.1 Limitations

As noted above, although we recruited a large sample from the UK Sikh population, it should be empahsised that this sample is not representative of whole UK Sikh community. Our sample is mostly young, university‐educated and computer literate. It should be noted that, although MH experiences are prevalent in our sample, there are other demographics not represented here. For example, those whose primary language is Punjabi may be more vulnerable to MH difficulties (Balan and Mahalingam 2015), such individuals are not well represented in the current study. It should also be noted that the emphasis of the ATMHP is on the psychological more so than the socio‐cultural aspects of shame. The UK Sikh community is collectivist in nature (Dhillon 2015) and hence the more individualistic emphasis of the ATMHP could mean that it does not adequately reflect the dimensions of shame that this community experiences.

This study was cross‐sectional in design and hence possible causal relations between key variables remain unclear, as noted above. Furthermore, mediation analysis itself has its limitations, for instance: it does not infer causality between variables and can be biased by the existence of unmeasured third variables or confounders. Future research should employ longitudinal or experimental designs to draw stronger inferences and enable more robust answers to such questions. It should be noted perhaps, that cross‐sectional online surveys have the advantage of being able to produce a large sample size, offering statistical power and confident conclusions about the reliability of inferential tests. They also confer the advantage of high levels of confidentiality, particularly important with sensitive topics like mental health and help‐seeking. Nonetheless, this method undoubtedly excludes sections of the population which are less computer literate. Future research should accommodate the need for culturally sensitive methodologies, developed in codesign with Sikh communities to capture a more representative sample.

## 5 Conclusion

UK Sikh respondents mostly had a biopsychosocial view of MH causes and treaments. Many reported that lack of awareness of services, stigma and shame were barriers to help‐seeking. Respondents tended to score high on most dimensions of the ATMHP. Having had MH problems was related to external family‐related shame, perceived negative community‐related attitudes to mental health and internal shame. Those who had had MH problems were more likely to say they would help someone else with MH problems. In line with social rank theory (Gilbert 1998, 2000), the link between having MH problems and feeling internal shame was partly explained by how much shame one would feel from the family/community if afflicted with MH problems, not merely by the awareness of negative family/community attitudes. These findings may help inform the design of culturally appropriate mental health services (Jimenez et al. 2012) for the UK Sikh community. It should also be remembered, that MH stigma/shame is widespread, transcending cultural and community boundaries. Hence, future research should purposely seek to reveal the diverse ways MH shame and stigma can affect communities across the world.

## Ethics Statement

The survey was created through Online Survey software developed by the authors' University. Ethical approval was granted by the host University's Faculty of Arts Ethics Committee (Ref 2018/19:11).

## Consent

None of the respondents were recruited on the basis of being patients.

## Conflicts of Interest

The authors declare no conflicts of interest.

## Peer Review

The peer review history for this article is available at https://www.webofscience.com/api/gateway/wos/peer-review/10.1002/jcop.70043.

## Data Availability

The data that support the findings of this study are openly available in Open Science Framework at https://osf.io/cg845/?view_only=b4b493c5008a49b6b1307c8e2d43e737.
